# A Biliary Behemoth: A Report of a Rare Case of a Giant Choledochal Cyst

**DOI:** 10.7759/cureus.109285

**Published:** 2026-05-20

**Authors:** Mahipal Mahipal, Tarun Tarun, Anupma Anupma, Dushyant K Yadav, Rishampreet Kaur, Praveen Yadav, Versha Versha, Chiranji Bhardwaj

**Affiliations:** 1 Department of General Surgery, Pandit Bhagwat Dayal Sharma Post Graduate Institute of Medical Sciences, Rohtak, IND; 2 Department of Anaesthesiology, Pandit Bhagwat Dayal Sharma Post Graduate Institute of Medical Sciences, Rohtak, IND; 3 Department of Surgery, Pandit Bhagwat Dayal Sharma Post Graduate Institute of Medical Sciences, Rohtak, IND

**Keywords:** congenital biliary anomaly, extrahepatic bile duct, giant choledochal cyst, obstructive jaundice, roux-en-y hepaticojejunostomy

## Abstract

Choledochal cysts are very uncommon congenital anomalies of the biliary tract, most commonly diagnosed in childhood. Adult presentation is uncommon and often atypical. Giant choledochal cysts, defined as cysts measuring more than 10 cm, are exceptionally rare and present significant diagnostic and surgical challenges.

A 19-year-old female presented with a two-month history of dull-aching abdominal pain, progressive right upper quadrant abdominal swelling, and features of obstructive jaundice. Physical examination revealed deep jaundice and a large, non-tender abdominal mass. Imaging studies, including ultrasonography, contrast-enhanced computed tomography, and magnetic resonance cholangiopancreatography, demonstrated a large cystic lesion communicating with the gallbladder and common bile duct, consistent with a Todani type IA choledochal cyst. Exploratory laparotomy revealed a massive extra-hepatic biliary cyst measuring approximately 20 × 25 × 28 cm and containing over 1.6 L of bile. Complete excision of the cyst along with cholecystectomy was performed, followed by Roux-en-Y hepaticojejunostomy for biliary reconstruction. Giant choledochal cysts in adults are rare and may mimic other intra-abdominal cystic lesions due to their size and mass effect on adjacent organs. Accurate preoperative diagnosis is crucial for appropriate surgical planning. Complete excision of the cyst with biliary reconstruction is mandatory to prevent complications such as cholangitis, biliary cirrhosis, pancreatitis, and malignant transformation. Giant choledochal cysts are an extremely rare presentation of congenital biliary disease in adults. Despite diagnostic difficulties, timely recognition and complete surgical excision with biliary reconstruction offer excellent outcomes and remain the cornerstone of management.

## Introduction

Choledochal cysts are rare congenital malformations of the biliary system characterised by cystic dilatation of the intra-hepatic ducts, extra-hepatic bile ducts, or both. In 1723, Vater and Ezler first described choledochal cyst [1,2]. Although typically diagnosed in childhood, choledochal cysts may occasionally present in adulthood, often with non-specific or atypical symptoms.

Giant choledochal cysts, defined as cysts exceeding 10 cm in diameter, represent an exceptionally rare subset, with few cases reported in the literature [3,4]. The exact aetiology remains unclear; however, an anomalous pancreaticobiliary junction (APBJ) has been implicated in approximately 30-70% of cases and is considered the acceptable pathogenic mechanism [5].

The incidence of choledochal cysts varies geographically, occurring in approximately 1 in 1,000 live births in Asian populations, compared with 1 in 100,000-150,000 in the United States of America and 1 in 2 million in the United Kingdom [6]. In 1959, Alonso-Lej et al. classified choledochal cysts into three types [7], a system later modified by Todani et al. in 1977 to include five types, with further sub-classification of type I lesions [8].

In adults, choledochal cysts are frequently asymptomatic, though complications such as hepatic abscess, biliary cirrhosis, biliary perforation and acute pancreatitis may occur and occasionally represent the initial presentation [9,10]. While small cysts are usually readily identified on imaging, giant choledochal cysts may compress adjacent organs, including the liver, pancreas, duodenum, and kidney, making preoperative diagnosis challenging and raising the possibility of alternative intra-abdominal cystic lesions. Accurate preoperative identification is crucial, as complete cyst excision with biliary reconstruction is mandatory to prevent long-term complications.

## Case presentation

An apparently well, 19-year-old female presented to the outpatient department with a two-month history of constant, dull-aching, mild-intensity, non-radiating abdominal pain associated with a progressively enlarging right hypochondrium abdominal mass. Her symptoms were accompanied by jaundice, characterised by yellowish discolouration of the eyes, dark urine, and clay-coloured stools. There was no history of diarrhoea, fever, vomiting or weight loss. She had no prior major illness, hospitalisation, or surgery reported.

On physical examination, the patient was moderately built in general condition, vitally stable and well nourished with a BMI of 20.9 kg/m^2^. The abdomen was distended with a palpable mass extending from the epigastrium to the umbilical region, crossing the midline. The mass was soft, non-mobile, without local rise of temperature, non-tender, and measured approximately 20 × 20 cm. The upper and lower margins of the mass could not be distinctly delineated. There were no visible dilated veins, and no palpable lymphadenopathy was detected. Patient’s laboratory parameters were within normal limits except an alkaline phosphatase (ALP) of 488 IU/mL, total bilirubin of 14.0 mg/dL and direct bilirubin of 7 mg/dL. Carcinoembryonic antigen (CEA) and carbohydrate antigen 19-9 (CA19-9) were within normal limits.

**Figure 1:**
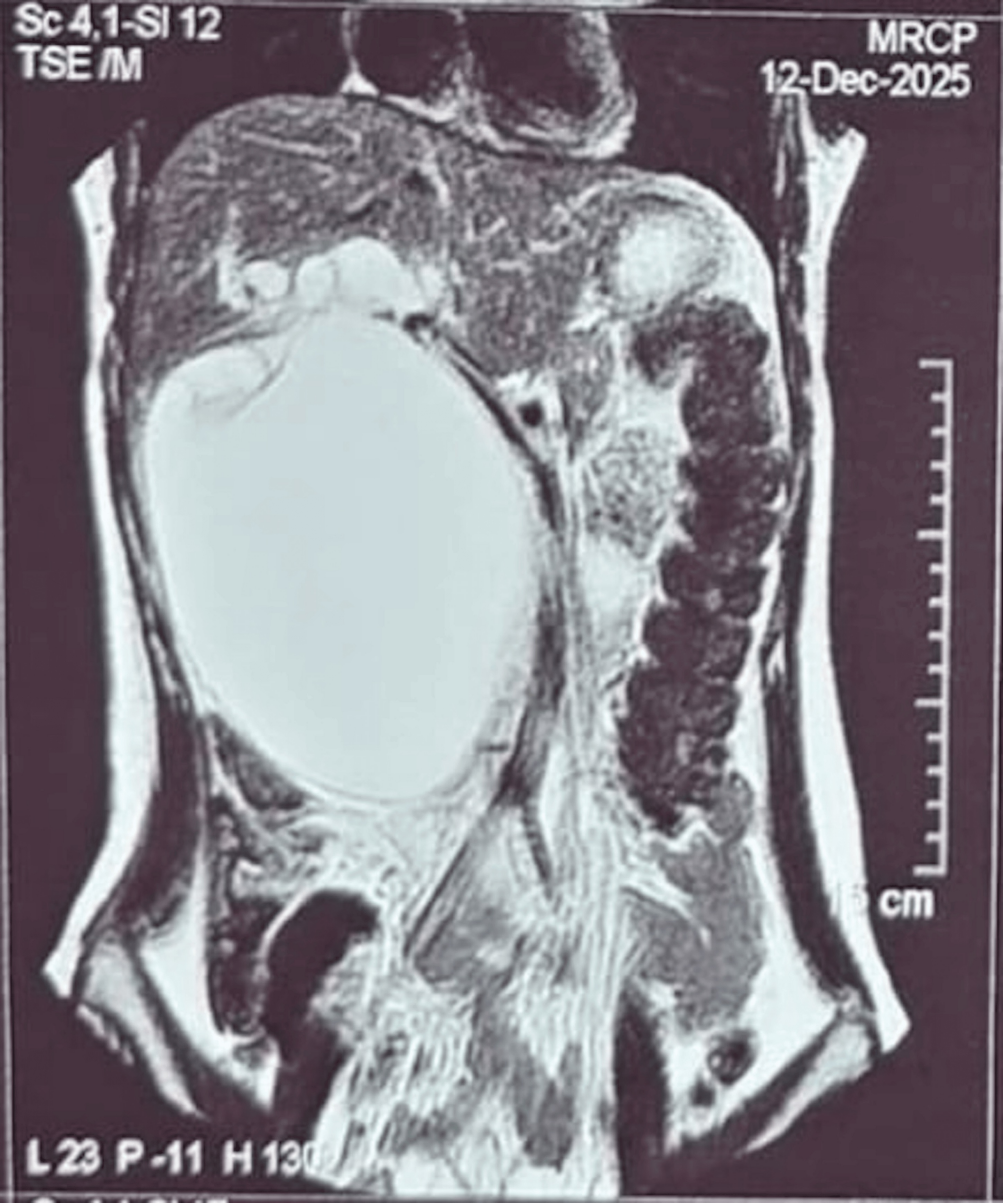
MRCP image showing a large choledochal cyst MRCP, magnetic resonance cholangiopancreatography.

Abdominal ultrasonography demonstrated a liver of normal size and echotexture, with mildly prominent intra-hepatic biliary radicles. The gallbladder was partially distended with an echo-free lumen. A large cystic lesion was identified in the gallbladder fossa. Contrast-enhanced computed tomography (CECT) of the whole abdomen and magnetic resonance cholangiopancreatography (MRCP) revealed a large cystic lesion measuring approximately 12 × 10 × 14 cm, without evidence of calcification, septations or solid components. The lesion was seen communicating with the gallbladder and involved the cystic duct and common bile duct, with associated mild-to-moderate intra-hepatic biliary radicle dilatation (Figure 1). These findings were suggestive of a type IA choledochal cyst. APBJ was not assessable in the MRCP.

The patient underwent an exploratory laparotomy through a modified Makuuchi incision. On entering the peritoneal cavity, there was no ascites and no evidence of lymphadenopathy. Intraoperative exploration revealed a markedly enlarged cystic mass extending from the liver to the hypogastric region. The gall bladder, duodenum and greater omentum were adherent to the cystic mass.

**Figure 2:**
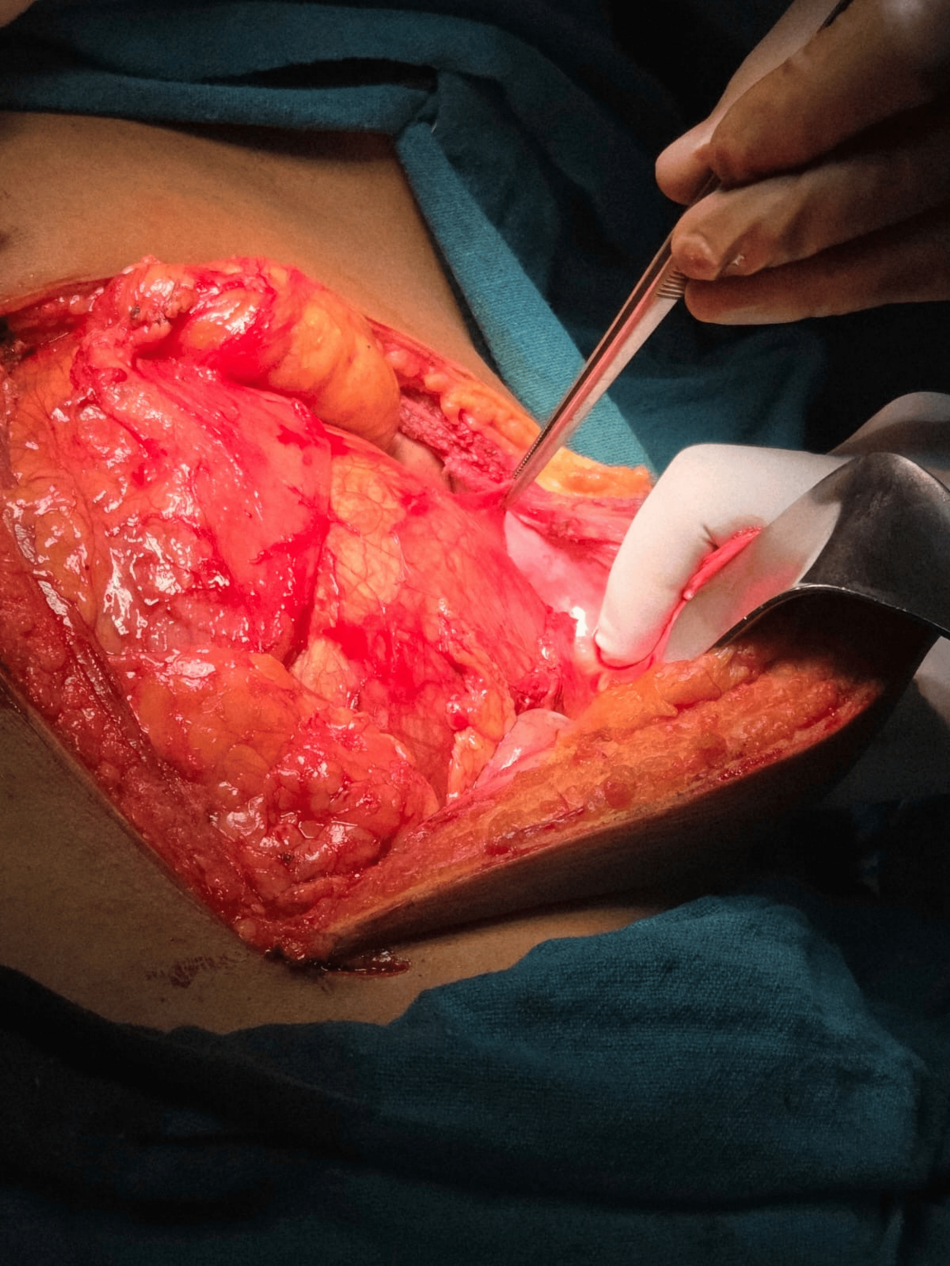
Intraoperative image showing a large choledochal cyst adhered to the omentum and duodenum

**Figure 3:**
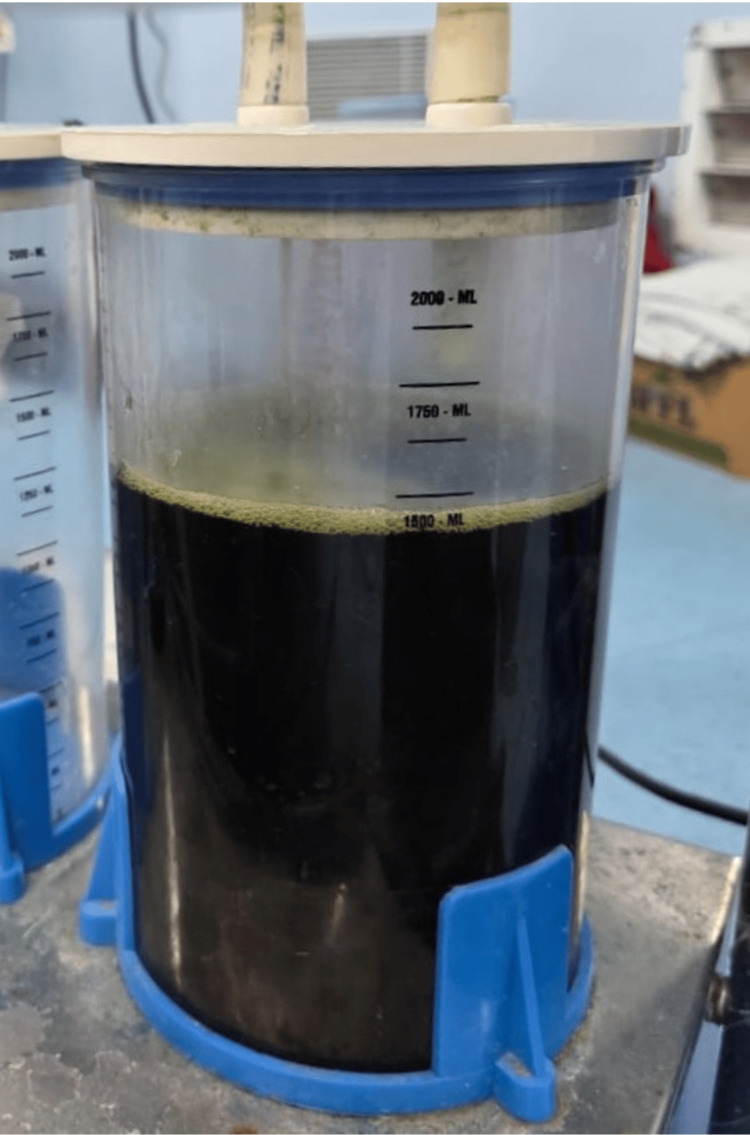
Image showing 1.5 L of greenish, thick and bilious fluid aspirated from the choledochal cyst

The greater omentum was adherent and draped over the anterior surface of the cyst, while the duodenum was stretched and adherent over its left anteromedial aspect (Figure 2). The pancreas was adherent to the posteromedial wall of the cyst. Following meticulous lateromedial dissection, these adhesions were carefully separated using blunt dissection and monopolar and bipolar cautery, without any discernible injury to the adjacent structures. This allowed adequate exposure of the lesion, which was identified as a massive diffuse extra-hepatic biliary cyst measuring approximately 20 × 25 × 28 cm, which was aspirated and reduced to the size 10 × 12 × 12 cm. The cyst predominantly involved the common bile duct and contained 1.5 L of greenish, thick, bilious fluid (Figure 3). The distal end of the cyst was blind ending, with no evidence of distal tapering. Intraoperative cholangiography was not performed.

The gallbladder was markedly distended, and the cystic duct formed the superolateral border of the cyst; therefore, an anterograde cholecystectomy was performed. The common hepatic duct and both right and left hepatic ducts were dilated. The cyst was transected approximately 1 cm proximal to the confluence of the common hepatic duct, followed by complete excision of the extra-hepatic biliary cyst.

**Figure 4:**
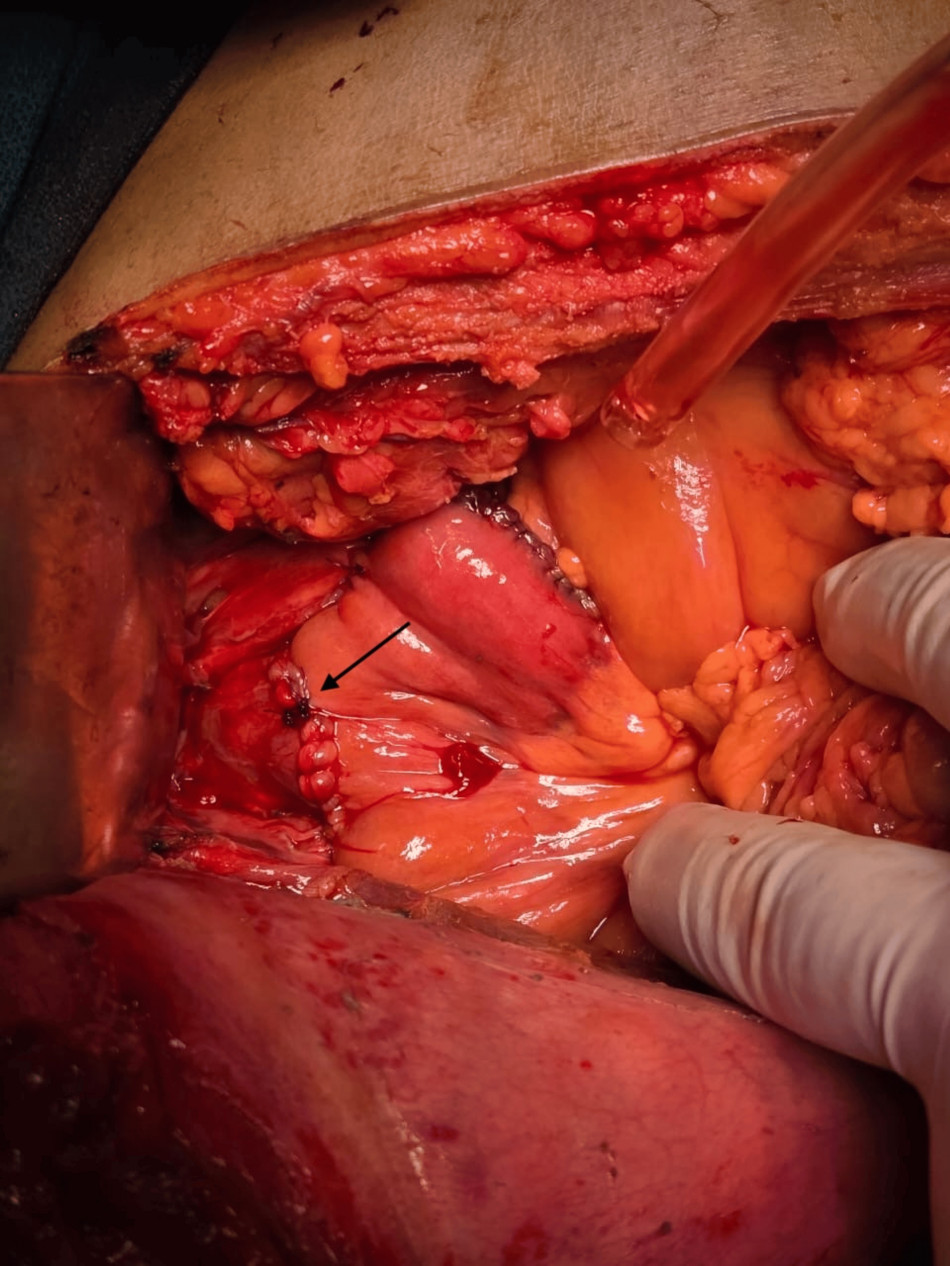
Intraoperative image showing Roux-en-Y hepaticojejunal anastomosis (arrow)

**Figure 5:**
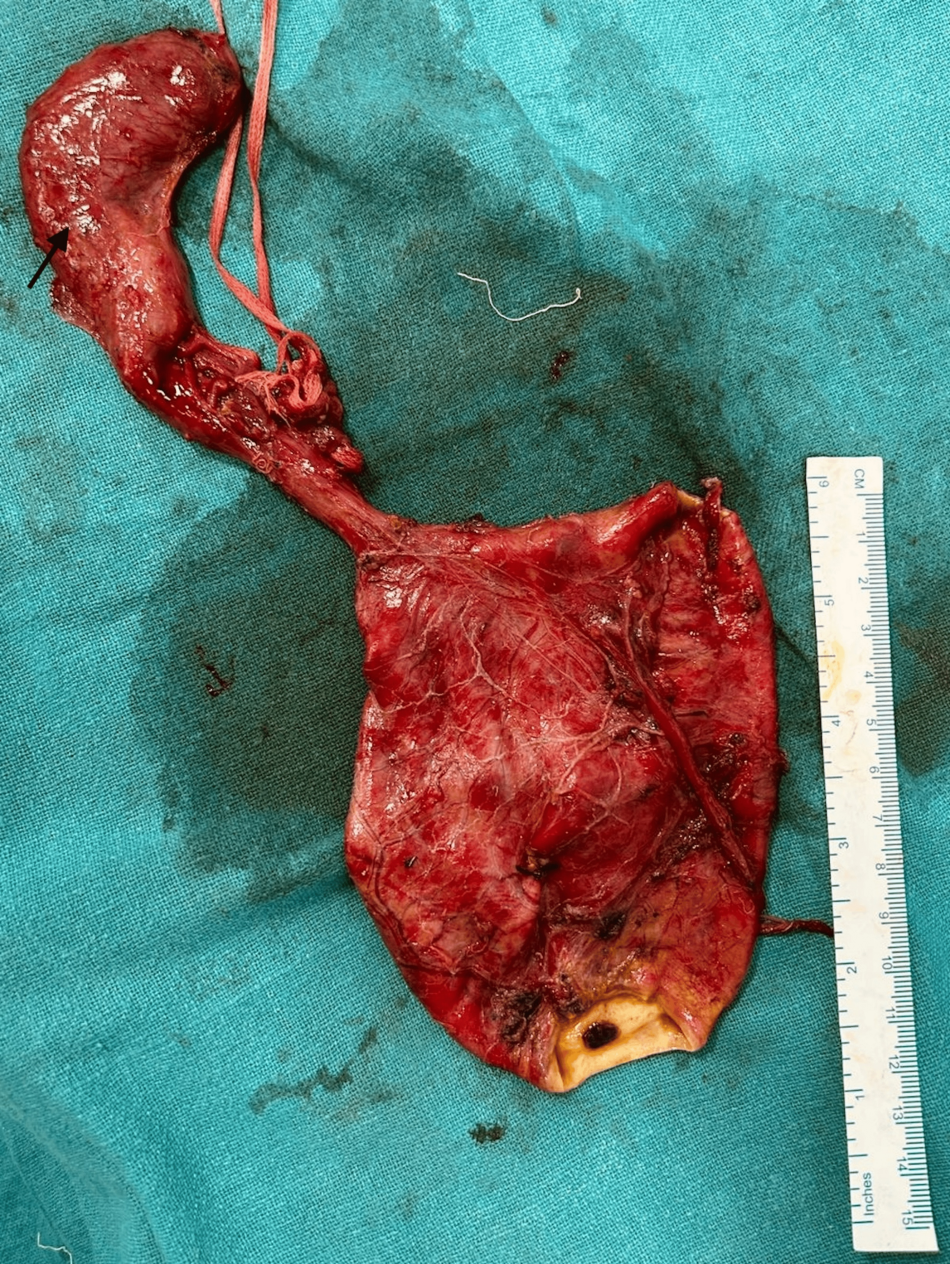
Image showing the specimen of choledochal cyst measuring 10x12 cm with the gall bladder (arrow)

A jejunal loop approximately 30 cm distal to the ligament of Treitz was mobilised, a defect was created on the left side of the middle colic vessels in the transverse mesocolon, and the mobilised limb was brought to the common hepatic duct stump through the defect. Biliary reconstruction was accomplished with a Roux-en-Y hepaticojejunostomy and jejunojejunostomy (Figure 4). A surgical drain was placed adjacent to the hepaticojejunostomy anastomosis. The specimen (Figure 5) was sent for histopathological examination. On the seventh post-operative day, the drain was removed, the patient’s jaundice was resolved, and the patient was discharged on the eighth post-operative day. On follow-up, the patient’s blood investigations were within normal limits, including liver function test, and the post-operative period was uneventful. Later, the histopathological report confirmed a choledochal cyst with chronic inflammatory changes and chronic cholecystitis in the gall bladder, with no evidence of dysplasia or malignancy.

## Discussion

Choledochal cysts are rare congenital malformations of the biliary system characterised by varying degrees of cystic dilatation of the intra-hepatic bile ducts, extra-hepatic biliary tree, or both [2]. Giant choledochal cysts, defined as cysts exceeding 10 cm in diameter, represent an exceptionally rare entity, with only very few cases reported in the literature [3,4,8].

The incidence of choledochal cysts shows marked geographic variation, occurring in approximately 1 in 1,000 live births in Asian populations, compared with 1 in 100,000-150,000 in the United States of America and 1 in 2 million in the United Kingdom [6]. The most widely accepted classification system is the Todani classification, which categorises choledochal cysts into five subtypes based on morphology, location and extent of involvement within the biliary tree. Type I cysts are limited to the extra-hepatic bile ducts and are further subdivided into type Ia (cystic dilatation of the common bile duct), type Ib (segmental or focal dilatation) and type Ic (fusiform dilatation). Types II and III represent an extra-hepatic bile duct diverticulum and choledochocele, respectively. Type IV is subdivided into two types: type IVa, involving both intra-hepatic and extra-hepatic bile ducts, and type IVb, involving multiple extra-hepatic dilatations only. Type V, also known as Caroli’s disease, involves isolated intra-hepatic bile duct dilatation. Type I and type IV cysts are the most commonly encountered forms in both paediatric and adult populations, accounting for approximately 50-80% and 15-50% of cases, respectively [11,12].

The most widely accepted aetiological theory for choledochal cyst formation is the presence of an APBJ [5]. In this anomaly, the common bile duct and the pancreatic duct unite just proximal to the sphincter of Oddi, allowing reflux and mixing of pancreatic and biliary secretions. The activation of pancreatic enzymes within the biliary tree leads to increased intra-ductal pressure, chronic inflammation, epithelial injury and progressive bile duct dilatation. Over time, these pathological changes may result in dysplasia and increase the risk of biliary tract malignancy [12,13].

Clinical presentation differs between children and adults. Children classically present with obstructive jaundice, whereas adults more commonly exhibit non-specific biliary or pancreatic symptoms, including right upper quadrant abdominal pain, jaundice, nausea, vomiting and fever [14]. The classic triad of jaundice, palpable abdominal mass and abdominal pain is uncommon, occurring in only approximately 20% of cases [11]. The patient described in this report presented with all three features of the classic triad, including a visibly apparent abdominal mass causing significant abdominal asymmetry and discomfort, highlighting the unusual nature of this presentation.

Abdominal ultrasonography is typically the initial imaging modality for evaluation of suspected choledochal cysts due to its availability and non-invasive nature. However, ultrasound has limitations in assessing the entire biliary tree because of its limited field of view. Cross-sectional imaging modalities, such as computed tomography (CT) and magnetic resonance imaging (MRI), provide superior anatomical detail and better delineation of large or complex cysts. MRCP is currently the preferred diagnostic modality because it is non-invasive, does not require contrast administration, and provides high-resolution visualisation of biliary anatomy and its relationship to adjacent structures [15].

In the present case, abdominal ultrasonography was used as a screening tool to identify the cystic lesion, while CT scan and MRCP were essential for confirming the diagnosis, assessing the extent of disease, and accurately classifying the lesion according to the Todani system. These imaging modalities enabled preoperative identification of a type IA choledochal cyst, which was subsequently confirmed intra-operatively.

Definitive management of choledochal cysts involves initial control of acute complications, followed by complete surgical excision of the cyst and restoration of biliary-enteric continuity. Total excision of the choledochal cyst with biliary reconstruction, either by Roux-en-Y hepaticojejunostomy or hepaticoduodenostomy, is currently the most widely accepted surgical approach [16]. In the present case, biliary reconstruction was achieved using a Roux-en-Y hepaticojejunostomy, with an anastomosis between the common hepatic duct and a jejunal loop approximately 30 cm distal to the ligament of Treitz.

Minimally invasive approaches, particularly laparoscopic excision, are increasingly being explored for the management of choledochal cysts, especially in paediatric patients, due to reduced postoperative pain and faster recovery [17]. However, laparoscopic surgery remains limited in many resource-constrained settings and is generally more suitable for smaller cysts, typically measuring less than 7 cm [18]. Given the massive size of the cyst in this patient and the presence of dense adhesions, an open surgical approach was considered the safest and most appropriate option.

## Conclusions

Giant choledochal cysts are an exceptionally rare congenital biliary abnormality in adults, and may mimic other large intra-abdominal cystic lesions, leading to diagnostic difficulty; only a limited number of cases have been reported worldwide. Accurate preoperative imaging plays a crucial role in establishing the diagnosis and planning surgical management. Complete cyst excision with appropriate biliary reconstruction remains the treatment of choice to achieve favourable outcomes and prevent long-term complications.
